# Unemployment and sickness allowance onset and prevalence: a longitudinal register-based study

**DOI:** 10.1186/s12889-026-27929-6

**Published:** 2026-05-28

**Authors:** Hanna Rinne

**Affiliations:** Rehabilitation Foundation Finland, PO BOX 39, Helsinki, 00411 Finland

**Keywords:** Unemployment, Sickness absence, Longitudinal study, Sickness allowance

## Abstract

**Introduction:**

Although unemployed people generally have poorer health than those who are employed, they are not more likely to receive sickness allowance. When they do, benefit periods tend to be longer, suggesting that non-take-up of sickness allowance may be more common among the unemployed. We examined how unemployment is associated with the onset and prevalence of sickness allowance.

**Methods:**

We used longitudinal register data on employed and unemployed residents aged 25–62 years in Finland during 2022–2023 (*N* = 1,774,257). We estimated random-effects (RE) and fixed-effects (FE) linear probability models and conditional FE logistic regression for dichotomous outcomes of sickness allowance onset and prevalence, and FE Poisson regression for the total number of sickness allowance days.

**Results:**

Among women, the probability of sickness allowance onset was slightly lower among the unemployed than among the employed (− 1.0 percentage points, 95% CI − 1.0 to − 0.9), In within-individual analyses, transitioning into unemployment was associated with a larger reduction (− 2.3 percentage points, 95% CI − 2.4 to − 2.1). Associations among men were similar but weaker. For sickness allowance prevalence among women, the probability of receiving sickness allowance was higher among the unemployed than among the employed (1.3 percentage points, 95% CI 1.2 to 1.5), whereas within-individual analyses indicated no clear change (− 0.2 percentage points, 95% CI − 0.5 to 0.1). However, the within-individual Poisson model showed that the total number of sickness allowance days was higher during unemployment (IRR 2.08, 95% CI 1.96 to 2.21). Results for men were broadly similar, although the within-individual models indicated a small reduction in the likelihood of receiving sickness allowance (− 0.5 percentage points, 95% CI − 0.7 to − 0.3).

**Conclusions:**

Unemployment is associated with the lower likelihood of entering sickness allowance, while its association with overall prevalence is weak. At the same time, unemployment is linked to a greater accumulation of sickness allowance days. These findings may reflect underutilization of sickness allowance among unemployed people. Reducing barriers to accessing sickness allowance for unemployed people would help to better identify work incapacity and ensure that those in need receive appropriate support.

## Introduction

Unemployed people form an important labour reserve, making it essential to monitor and support their work ability. In 2025, the unemployment rate was 5.9% in the EU and 10.2% in Finland [1]. Maintaining the health and work ability of unemployed people is also vital for social justice, reducing socioeconomic health inequalities, and lowering healthcare costs. Undetected illnesses can worsen over time. In Nordic welfare states, unemployment benefits are paid to those able to work and willing to accept job offers. If an unemployed person becomes ill, they are expected to apply for sickness allowance instead. However, unlike employees, unemployed people are not required to report sick leave, which may lead to eligible individuals not claiming sickness allowance. Across Europe, stricter access to disability pensions and stronger activation policies have created a group of unemployed people with health problems who fall between systems [2]. As welfare states move toward workfare-oriented activation [3, 4], more individuals with complex health and social issues enter employment services. With tighter conditionality, sickness benefits are becoming more important.

Several studies have shown that unemployed people have poorer physical and mental health than employed people. Those who become unemployed tend to have worse health on average than employed people, but unemployment itself may also have adverse effects on health, especially on mental health [5–10]. Unemployment may negatively affect living conditions, income, social relationships, access to healthcare, and health behaviours [11]. The harmful health effects have been shown to increase with the duration of unemployment [9, 12].

There is little research on transitions into sickness allowance among the unemployed, as most studies focus on employees. A Finnish study [13] found that although unemployed people had fewer sickness absence spells than employees, they had clearly more sickness absence days. Because employment status was defined based on the previous year, individuals were not necessarily unemployed when entering sickness allowance. Another study [14] showed that among women, sickness absence risk due to mental disorders was similar for the unemployed and most other socioeconomic groups, while among men the risk was highest for the unemployed. Unemployment has also been linked to an increased risk of work disability and sickness absence among young people [15, 16]. A common feature of these studies is that unemployment was defined before follow-up, rather than examining transitions into sickness benefits during ongoing unemployment. A German survey study [17] further found that among the unemployed, men, older individuals, and those with lower education were more likely to receive sickness absence even after adjusting for health factors.

In Finland, sickness allowance provides income compensation for individuals aged 18–67 who are temporarily unable to do their usual work due to illness [18]. Both employed and unemployed people are all eligible for sickness allowance. The benefit is paid after a waiting period consisting of the first day of illness plus nine working days and can be received for up to 300 working days within a single spell of illness. A physician’s certificate is required, and eligibility is based on assessed work incapacity rather than the mere presence of illness. The Social Insurance Institution (Kela) evaluates work capacity with reference to the individual’s most recent job, drawing on a certificate from the treating physician and, when needed, the expertise of insurance medicine specialists. After reaching the 300 day maximum, an individual must be fit for work for at least one year before becoming eligible again for the same illness.

There are several reasons why unemployed and employed individuals differ in their transitions to sickness allowance, many of which relate to the broader phenomenon of *non-take-up of social benefits*, where eligible individuals do not claim the benefits to which they are entitled [19]. According to van Oorschot, non-take-up can be understood at three levels, client, administrative, and policy design [20], further elaborated by van Mechelen and Janssens [21].

The key *client level factor* is the need. Unemployed people generally have poorer health than the employed, increasing the risk of sickness absence. Worsening health raises the likelihood of entering sickness allowance, and prolonged ill-health is associated with higher prevalence. Unemployed people have no obligation to report sickness, which may lead them not to apply for sickness allowance even when eligible. In contrast, employees must report sickness from the first day and typically need a medical certificate within a few days.

According to the non-take-up framework, clients face several types of costs: information and process costs, psychological and social costs, and behavioural barriers [21]. These are particularly pronounced for unemployed individuals. Unemployed people may be unaware they are eligible for sickness allowance [22] or may not recognise their need for it [23]. The application process is complex and bureaucratic, and information and process costs are often far higher for unemployed claimants [22]. Psychological and social costs, such as fear of stigma, may also discourage claiming [21], although sickness does not necessarily add stigma for the unemployed [24]. Social network effects may further influence decisions among the unemployed more strongly than those in employment.

Incentives also differ markedly. While employed individuals clearly benefit from sickness absence as a relief from work and income compensation, the short-term gains for unemployed people are less obvious because they already receive financial support. For them, the main advantages of sickness allowance are long-term, such as its role in assessing rehabilitation and disability pension needs. Because short-term motivations often dominate decision-making [21], unemployed individuals may not perceive immediate benefits from claiming. A benefit of sickness allowance is that unemployed recipients are exempt from participating in employment services during the allowance period. Activation measures may even direct individuals toward sickness allowance, although participation can also improve health [25]. Moreover, sickness allowance does not reduce one’s maximum entitlement to earnings-related unemployment benefits in Finland. The duration of the illness and the benefit level further influence the incentives to claim sickness allowance [21].

*Administrative barriers* form another axis of non-take-up. The degree and quality of information provided to applicants, the user-friendliness of procedures, and organisational capacity all influence whether eligible individuals claim sickness allowance [21].

In Finland, employees usually have rapid access to occupational health services, where medical certificates are easy to obtain. Unemployed people, by contrast, rely on public healthcare with longer waiting times and more difficult access. Lower overall service use among the unemployed [26, 27], reduced healthcare use after job loss [28], and higher unmet need [23] all signal barriers to obtaining the documentation required for an application. Moreover, physicians issuing medical certificates may be unaware that an unemployed patient may also need the benefit, and many lack sufficient knowledge of social insurance [29].

There are also differences in how work capacity is assessed. For unemployed individuals, work capacity is evaluated in relation to their previous job or, after prolonged unemployment, to other jobs for which they are qualified. For employees, by contrast, work capacity is typically assessed by an occupational health specialist with reference to their current job. Physicians consider assessing the work ability of unemployed people more difficult than assessing that of the employed [29–32]. Overall, physicians in Finland and other Nordic countries often find sickness certification challenging [29–32], with occupational health physicians reporting the fewest difficulties [29]. They also struggle with evaluating how functional limitations affect work ability, determining the appropriate duration of sickness absence, and making recommendations for rehabilitation [29–32].

This difference makes the application process considerably more user-friendly for employed individuals. The internal organisation of the agencies responsible for policy delivery further shapes the experience: At Kela, heavy workloads, strict processing targets, and delays due to incomplete certificates further complicate the process [22]. Long processing times and administrative backlogs [22] can lead to distrust and discourage applying, particularly when rejection rates for unemployed applicants based on work ability assessments are several times higher than for employees [I. Hiljanen, Kela, personal communication, January 2026].

Sickness allowance is a key indicator of work incapacity and is used to assess rehabilitation needs and transitions to disability pension. Possible underutilization of sickness allowance among the unemployed people therefore hampers the identification of individuals who are unable to work and who might benefit from healthcare services and rehabilitation [33]. Social inequalities may widen further, if social policies primarily reach those who are already somewhat better positioned, in this case, the employed [19]. Therefore, it is essential to examine more closely how unemployed people use sickness allowance. Different measures of sickness absence capture distinct dimensions of the phenomenon [34]. The aim of this study is to examine 1) how unemployment is associated with the onset of sickness allowance, and 2) how unemployment is associated with the prevalence of sickness allowance.

## Methods

### Data

We used the individual-level register data covered all individuals aged 16–65 residing in Finland at the end of 2021, drawn from the registers of Statistics Finland. These data were linked with variables covering the period 2019–2023. Information on sickness allowance spells was obtained from the registers of the Social Insurance Institution of Finland, while employment spells were derived from the Finnish Centre for Pensions and unemployment spells from Statistics Finland. Socio-demographic background variables were obtained from Statistics Finland. Data on entitlement to specially reimbursed medication were derived from the Social Insurance Institution and visits in specialised healthcare from Finland Care Register for Health Care.

### Study population

**Fig. 1:**
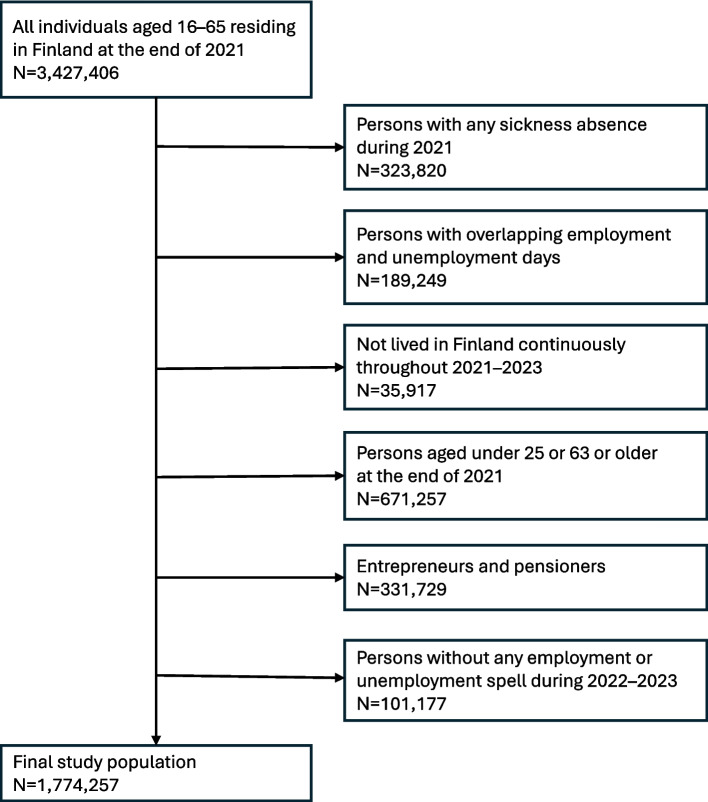
Flow chart of the study population

The study population consisted of all individuals aged 16–65 residing in Finland at the end of 2021 (Fig. 1). We excluded all persons with any sickness allowance during 2021 to eliminate issues related to maximum benefit duration, as a one-year consecutive non-use period resets the 300-day sickness allowance counter. Individuals with overlapping employment and unemployment days were also removed, as they were likely receiving adjusted unemployment benefits alongside part‑time earnings.

The analytic sample was further restricted to those who lived in Finland continuously throughout the 2021–2023 and who were aged 25–62 at the end of 2021. The lower age limit of 25 was chosen because many younger individuals are still students, while the upper limit ensures that participants had not yet reached the earliest possible old-age pension age of 63. We also excluded entrepreneurs and pensioners at the end of 2021, as pensioners are not eligible for sickness allowance and the application process for entrepreneurs differs from that of employees. Finally, we included only individuals who experienced at least one employment or unemployment spell during 2022–2023. The final number of study population was 1,774,257.

### Outcomes

Sickness absence was measured as the recipiency of sickness allowance, which covers sickness absences lasting more than 10 days. Each sickness allowance episode included both a start and an end date.

We calculated, for each calendar quarter (three-month period), (i) the number of new sickness allowance spells starting in that quarter and (ii) the total number of sickness allowance days accumulating within that quarter. Based on these measures, we constructed two dichotomous outcomes.

Sickness allowance onset was defined as having at least one new sickness allowance spell start during the quarter (yes/no). Sickness allowance prevalence was defined as having any sickness allowance days during the quarter (yes/no), regardless of whether the spell started in the same quarter or earlier. Prevalence therefore captures the proportion of individuals who were on sickness allowance at any point during the quarter.

In addition to these binary outcomes, we analysed sickness allowance as continuous measures, including the number of new sickness allowance spells and the total number of sickness allowance days per quarter.

### Exposures

Employment and unemployment spells were constructed using daily register data by combining consecutive periods, allowing interruptions of up to two days. Because unemployment benefits and sickness allowance cannot be received simultaneously, unemployment spells were merged with any immediately subsequent sickness allowance spells, interruptions of up to two days were allowed. These combined unemployment–sickness allowance periods were classified as unemployment.

For each calendar quarter, we calculated the total number of employment and unemployment days. Individuals were classified as employed if they had at least 90 employment days during the quarter and as unemployed if they had at least 90 unemployment days. Quarters not meeting either criterion were excluded. Consequently, the analytic sample included only quarters with stable employment or stable unemployment throughout the entire quarter.

Labour market status during sickness allowance was determined based on the preceding days. Sickness allowance outcomes were measured within the same quarter as employment status. By restricting the analysis to quarters with stable exposure status, employment status necessarily preceded any sickness allowance onset occurring within the quarter. Quarters with mixed labour market statuses or transitions between employment and unemployment were excluded by design.

The analysis therefore compared sickness allowance outcomes between quarters of stable employment and quarters of stable unemployment. Other labour market states, such as parental leave, studies or inactivity, were not included.

### Covariates

Analyses were conducted separately by sex, as both unemployment and sickness allowance use have been shown to differ between women and men [14, 35].

We accounted for the following background variables measured at the end of 2021: age, origin, education, living alone, home ownership, long-term disease, visit days in specialised healthcare during 2021 and municipality type. Long-term disease was defined based on entitlement to specially reimbursed medication [36]. Age, socioeconomic factors, and morbidity have been shown to be associated with both unemployment and sickness absence [13, 35]. The categories for all variables are presented in Table 2.

### Statistical analysis

We estimated the analyses using a linear probability model (LPM), which models the probability of a binary outcome as a linear function of the predictors. The model has several advantages. First, the outcome is relatively rare, and conditional fixed-effects logistic regression would exclude a substantial share of individuals with no within-individual variation in the dependent variable. The use of an LPM therefore allows us to retain the full sample. Second, the LPM provides easily interpretable coefficients that represent absolute percentage-point differences in outcome probabilities. Third, although the LPM can in principle generate predicted values outside the 0–1 range, this does not bias coefficient estimates, and statistical inference remains valid when using robust, cluster-adjusted standard errors.

We estimated both random-effects (RE) and fixed-effects (FE) specifications. The RE models allow the inclusion of time-invariant covariates, whereas the FE models control for all unobserved time-invariant individual characteristics.

We first fitted RE models adjusted for age and period (Model 1), followed by models adjusted for all background characteristics: age, period, origin, education, living alone, home ownership, long-term illness, and municipality type (Model 2). All covariates were included as categorical variables.

Individual fixed-effects LPMs were estimated to examine within-person changes in the likelihood of receiving sickness allowance across employment and unemployment periods (Model 3). These models estimate within-person associations between changes in employment status and the likelihood of receiving sickness allowance while accounting for time-invariant individual characteristics.

In addition, we estimated conditional fixed-effects logistic regression models (Model 4). Finally, outcomes were also analysed as continuous measures using fixed-effects Poisson regression with robust standard errors to account for overdispersion (Model 5).

The identifying samples differ between the models. While fixed-effects LPMs include all individuals in the study population, coefficient estimation relies on within-person variation in the exposure, such that the unemployment coefficient is identified by individuals who experience changes in employment status over time. In contrast, conditional fixed-effects logit and Poisson models are restricted to individuals with within-person variation in the outcome.

### Sensitivity analysis

We conducted subgroup analyses by educational level (basic vs. tertiary education) and age (25–34 and 55–62 years) to assess whether the association between unemployment and sickness allowance differed across population groups. Education- and age-stratified analyses were performed using the same modelling strategies as in the main analyses. In addition, we conducted sensitivity analyses including entrepreneurs and tested alternative definitions of employment and unemployment, defining both states as lasting at least 30 days within a period.

## Results

### Descriptive statistics

Overall, 18% of women and 11% of men had sickness allowance days at some point of the follow-up. 11% of women and 14% of men had unemployment periods (Table 1).

**Table 1:** Descriptive characteristics of the study population

Characteristics	Women	Men
N		
Individuals	878,518	895,739
Quarterly observations	7,028,144	7,165,912
% of observations		
Unemployed	4.7%	7.0%
Sickness allowance onset	3.0%	1.8%
Sickness allowance prevalence	4.1%	2.6%
Mean number		
Sickness allowance onset	0.03	0.02
Sickness allowance days	1.39	0.95
% of individuals		
Unemployed	10.6%	13.9%
Sickness allowance	17.9%	11.2%

Table 2 presents the study population composition, the number of transitions between employment and unemployment, and the individuals contributing to the fixed-effects analyses.

**Table 2:** Study population and variation in employment transitions

Number of individuals	
Total number	1,774,257
Always employed	1,590,905
Always unemployed	106,932
Switching between employment and unemployment	76,420
Number of transitions between consecutive observations	
Employment-to-unemployment	35,265
Unemployment-to-employment	9,552
Number of individuals contributing within-individual variation	
Onset (FE logit)	16,237
Onset (FE Poisson)	16,237
Prevalence (FE logit)	16,235
Prevanlence (FE Poisson)	16,237

Transitions are defined between consecutive observed periods. Missing information on employment status may reduce the number of observed transitions and the within-person variation contributing to the fixed-effects analyses

The study population showed largely similar distributions for women and men (Table 3). More than half were under 45 years old of age, and one tenth were of foreign origin. Compared to men, women were more highly educated and less likely to live alone. Two thirds owned their home, one fifth had a long-term disease, and three quarters resided in an urban-type municipality.

**Table 3:** Distribution of background factors in study population in 2021 (percent of individuals)

	Women	Men
Background factor	%	%
Age		
25–34	27	29
35–44	28	29
45–54	25	24
55–62	20	18
Origin		
Finnish	91	90
Foreign	9	10
Education		
Basic	8	13
Secondary	36	47
Tertiary	56	40
Living alone		
No	79	69
Yes	21	31
Home ownership		
No	31	34
Yes	69	66
Long-term disease		
No	83	85
Yes	17	15
Visit days in specialised healthcare		
0	54	69
1	17	13
2–3	14	10
4–9	11	6
10-	4	2
Municipality type		
Urban	76	77
Semi-urban	14	13
Rural	10	10
Total %	100	100
Total N	878,518	895,739

### Models

Among women, the probability of sickness allowance onset was modestly lower in unemployed women as compared to employed women (− 1.0 percentage points in the fully adjusted RE LPM model) (Table 4). In the within‑individual analysis, becoming unemployment was associated with 2.3 percentage points lower probability of sickness allowance onset as compared to when one was employed. These findings were consistent with results from the conditional within‑individual logistic regression (OR 0.48) and the within‑individual Poisson model for the number of new sickness allowance episodes (IRR 0.49).

**Table 4:** Unemployment and sickness allowance onset among women and men

	Model 1	Model 2	Model 3	Model 4	Model 5
	RD	RD	RD	OR	IRR
Women					
Employed	0.000	0.000	0.000	1.00	1.00
Unemployed	− 0.005	− 0.010	− 0.023	0.48	0.49
95% CI	(− 0.006, − 0.004)	(− 0.010, − 0.009)	(− 0.024, − 0.021)	(0.45, 0.51)	(0.46, 0.52)
rho	0.0765	0.0718	0.1973		
N	878,518	878,518	878,518	148,616	148,703
Obs	6,407,284	6,407,284	6,407,284	1,119,923	1,120,126
Men					
Employed	0.000	0.000	0.000	1.00	1.00
Unemployed	− 0.000	− 0.005	− 0.017	0.46	0.45
95% CI	(− 0.001, 0.000)	(− 0.006, − 0.005)	(− 0.019, − 0.016)	(0.43, 0.49)	(0.42, 0.48)
rho	0.0853	0.0814	0.2028		
N	895,739	895,739	895,739	92,726	92,790
Obs	6,595,091	6,595,091	6,595,091	695,003	695,154

*RD* risk differences, *OR* odds ratios and *IRR* incidence rate ratios

Model 1: RE LPM model adjusted for age

Model 2: RE LPM model adjusted for all

Model 3: FE LPM model

Model 4: FE logistic regression model

Model 5: FE Poisson regression model

Among men, the LPM estimates were directionally similar but smaller in magnitude than those observed among women. In turn, the results from the logistic and Poisson regression models did not differ between men and women.

The association between unemployment and sickness allowance prevalence differed from that observed for onset. Among women, sickness allowance was more common among the unemployed than among the employed, with a 1.3 percentage point higher prevalence in the fully adjusted between-individual model (Table 5). However, within-individual analyses suggested that becoming unemployed was associated with a slight reduction in the probability of receiving sickness allowance. At the same time, the total number of sickness allowance days was higher during periods of unemployment (IRR 2.08). The corresponding results for men were very similar to those observed among women.

**Table 5:** Unemployment and sickness allowance prevalence among women and men

	Model 1	Model 2	Model 3	Model 4	Model 5
	RD	RD	RD	OR	IRR
Women					
Employed	0.000	0.000	0.000	1.00	1.00
Unemployed	0.018	0.013	− 0.002	0.92	2.08
95% CI	(0.017, 0.020)	(0.012, 0.015)	(− 0.005, 0.001)	(0.87, 0.97)	(1.96, 2.21)
Rho	0.2033	0.1974	0.2956		
N	878,518	878,518	878,518	148,902	149,938
Obs	6,407,284	6,407,284	6,407,284	1,122,408	1,125,997
Men					
Employed	0.000	0.000	0.000	1.00	1.00
Unemployed	0.017	0.011	− 0.005	0.81	1.99
95% CI	(0.016, 0.018)	(0.10, 0.012)	(− 0.007, − 0.003)	(0.76, 0.85)	(1.87, 2.12)
Rho	0.2211	0.2162	0.3096		
N	895,739	895,739	895,739	93,089	93,959
Obs	6,595,091	6,595,091	6,595,091	697,977	700,912

*RD* risk differences, *OR* odds ratios and *IRR* incidence rate ratios

Model 1: RE LPM model adjusted for age

Model 2: RE LPM model adjusted for all

Model 3: FE LPM model

Model 4: FE logistic regression model

Model 5: FE Poisson regression model

In the onset analyses, adjustment for education and the number of specialist healthcare visit days led to the largest increase in the estimated differences between unemployed and employed people, for both women and men (Table 6). Conversely, in the prevalence analyses, these same adjustments explained the largest share of the differences.

**Table 6:** Unemployment and sickness allowance onset and prevalence and their associations with background factors, risk differences (RD)

	Model 1 age	Model 1a origin	Model 1b education	Model 1c living alone	Model 1d home ownership	Model 1e long-term disease	Model 1f health care use	Model 1 g municipality type
Onset								
Women								
Employed	0.000	0.000	0.000	0.000	0.000	0.000	0.000	0.000
Unemployed	− 0.005	− 0.004	− 0.007	− 0.005	− 0.006	− 0.006	− 0.007	− 0.005
95% CI	(− 0.006, − 0.004)	(− 0.004, − 0.003)	(− 0.008, − 0.007)	(− 0.006, − 0.004)	(− 0.007, − 0.006)	(− 0.006, − 0.005)	(− 0.008, − 0.006)	(− 0.005, − 0.004)
Men								
Employed	0.000	0.000	0.000	0.000	0.000	0.000	0.000	0.000
Unemployed	− 0.000	− 0.000	− 0.003	− 0.001	− 0.001	− 0.001	− 0.002	− 0.000
95% CI	(− 0.001, 0.000)	(− 0.001, 0.000	(− 0.003, − 0.002)	(− 0.001, − 0.000	(− 0.002, − 0.001)	(− 0.001, − 0.000)	(− 0.003, − 0.002)	(− 0.001, 0.000)
Prevalence								
Women								
Employed	0.000	0.000	0.000	0.000	0.000	0.000	0.000	0.000
Unemployed	0.018	0.020	0.016	0.018	0.017	0.017	0.016	0.019
95% CI	(0.017, 0.020)	(0.019, 0.022)	(0.014, 0.017)	(0.017, 0.019)	(0.015, 0.018)	(0.016, 0.019)	(0.014, 0.017)	(0.017, 0.020)
Men								
Employed	0.000	0.000	0.000	0.000	0.000	0.000	0.000	0.000
Unemployed	0.017	0.017	0.014	0.016	0.016	0.016	0.014	0.017
95% CI	(0.016, 0.018)	(0.016, 0.018)	(0.013, 0.015)	(0.015, 0.017)	(0.015, 0.017)	(0,015, 0.017)	(0.013, 0.015)	(0.016, 0.018)

Model 1 RE LPM model adjusted for age

Models 1a-1g RE LPM model adjusted for age and the respective background factor

### Subgroup and sensitivity analyses

We conducted analyses stratified by educational attainment (basic vs. tertiary education; results not shown). Overall, education-stratified analyses revealed only modest differences. In the onset analyses, between-individual models indicated a slightly larger reduction in the probability of sickness allowance onset among individuals with basic education than among those with tertiary education. Within-individual models among men showed a similar pattern. In the prevalence analyses, the association of unemployment also appeared slightly stronger among individuals with lower educational attainment.

Age-stratified analyses (25–34 vs. 55–62 years; results not shown) indicated that age did not modify the within-individual association of unemployment on sickness allowance onset. In prevalence analyses, unemployment was associated with reduced sickness allowance use among younger individuals, but with longer sickness allowance durations particularly among older individuals.

We also conducted a sensitivity analysis including entrepreneurs. The results were very similar to those obtained when entrepreneurs were excluded. In addition, we tested alternative definitions of employment and unemployment. When defining both states as lasting at least 30 days per period, the results remained directionally consistent, although the association of unemployment was slightly smaller.

## Discussion

Unemployment is associated with the lower likelihood of sickness allowance onset. Its relationship with sickness allowance prevalence is more complex and appears to be minimal. By contrast, unemployment is associated with a higher overall number of sickness allowance days.

The between-individual models examining differences across employed and unemployed people showed that sickness allowance onset was slightly lower among unemployed people, while prevalence was higher. In contrast, within-individual analysis showed that becoming unemployed was associated with a somewhat larger reduction in the probability of entering sickness allowance and little or no change in the likelihood of receiving it, but with a higher number of sickness allowance days during unemployment periods.

The differing results between the models likely reflect selective processes and unobserved differences between employed and unemployed individuals, as well as the influence of time-invariant individual characteristics accounted for in the within-individual models. Within-individual estimates apply to a selected group of individuals and should not be interpreted as population-average effects. Although the fixed-effects LPM includes all individuals, the unemployment coefficient is identified by those who experience changes in employment status over time, while the conditional fixed-effects models are further restricted to individuals with within-person variation in sickness allowance. As such changes are relatively limited, the estimates are driven by a relatively small and selected subgroup of the study population. Moreover, while fixed effects account for time-invariant individual characteristics, they do not control for time-varying factors such as changes in health, healthcare use, or job loss circumstances. For example, unemployment is known to be associated with adverse mental health outcomes [7, 10] and may also reduce the use of healthcare services [28], which can influence sickness allowance onset.

The pattern of reduced onset, minimal changes in prevalence, and increased sickness allowance days suggests a selective process in which fewer unemployed people enter sickness allowance, but those who do experience longer benefit periods. The results underscore the importance of examining sickness allowance using multiple outcome measures [34], as relying on a single indicator may obscure important differences between the onset of sickness allowance and the accumulation of sickness allowance days.

These findings are consistent with earlier studies showing that unemployed individuals are less likely to take up sickness allowance, although those who do tend to receive benefits for longer periods [13]. They are also in line with the broader non-take-up literature, which shows that take-up rates are substantially lower among eligible individuals whose expected benefit duration is relatively short [21]. When anticipated gains are perceived as small, individuals may be less inclined to invest time and effort in gathering information, understanding eligibility conditions, and navigating application procedures [21].

Among unemployed individuals who do enter sickness allowance, longer durations may indicate that those who apply are, on average, in poorer health than their non‑applying counterparts. Alternatively, sickness allowance periods may be prolonged due to physicians’ tendency to issue longer sick leaves than strictly necessary, particularly when diagnostic examinations or treatment pathways are delayed [30]. Such delays may be more common among unemployed individuals, as their access to timely healthcare and rehabilitation is often more limited than that of employed individuals [23, 33].

The results suggest that educational differences in the association between unemployment and sickness allowance are modest. Unemployment tends to be somewhat more strongly associated with the onset and prevalence of sickness allowance among individuals with lower education, particularly among men with respect to sickness allowance onset. Differences by education level may reflect unequal access to information as well as greater difficulties in navigating the application process.

The relationship between unemployment and sickness allowance also varies by age. Analyses of sickness allowance prevalence and its number show that unemployment is linked to lower use particularly among younger individuals. Among older individuals, unemployment is more closely associated with a greater accumulation of sickness allowance days, which may reflect more severe or long-lasting health problems and a stronger tendency to exit the labour market for health-related reasons at later working ages.

Unemployment may involve varying activation requirements, which could influence whether individuals remain on unemployment benefits or transition to sickness allowance when ill. If activation demands are substantial, illness may encourage transitions out of unemployment, whereas with more limited demands, individuals may remain administratively unemployed despite illness. Activation measures may also have both positive and negative effects on wellbeing, for example by providing structure and social contact but also by reducing autonomy or creating mismatches with individual capacities [37]. Participation in such measures has also been linked to improved health [25]. As our data did not include information on activation obligations and only limited information on labour market programme participation, these mechanisms cannot be directly assessed.

Differences between unemployed and employed people may also reflect differences in the diagnostic composition of sickness allowance. Previous research shows that unemployment is associated with mental health problems [6, 7, 10]. A higher proportion of unemployed individuals apply for sickness allowance for mental health reasons than employed individuals, and such diagnoses are also linked to higher rejection rates [I. Hiljanen, Kela, personal communication, January 2026].

The results may reflect underutilization of sickness allowance among relatively healthier unemployed individuals with some labour market attachment. Although unemployment may increase the risk of sickness absence through its adverse effects on health, the administrative onset of sickness allowance may appear lower among unemployed people due to higher levels of non‑take‑up and barriers encountered at both the client and administrative levels. Not only health status but also societal definitions, organisational routines and individual interactions influence which unemployed individuals are classified as sick and granted sickness allowance [17]. However, the present analysis does not allow us to distinguish between alternative mechanisms underlying this pattern. Consequently, benefit registers may systematically underestimate the true prevalence of work disability among the unemployed. These findings underscore the complexity of health-related behaviour during unemployment and the need for careful methodological consideration in future research.

Prolonged sickness absence may lead to deeper labour market marginalisation [38, 39], particularly if the individual does not receive vocational training, rehabilitation, or other forms of support to address the underlying work disability during the sickness absence period. Unemployment increases the risk of disability pension [16, 40, 41], although rehabilitation applications among the unemployed [42] as well as disability pension applications [43] are more frequently rejected.

At the same time, access to disability pensions has been tightened, and the obligations of unemployed individuals to participate in activation measures have increased. This has created a group of unemployed people with health problems who fall between systems [2]. Under tighter conditionality, sickness benefits have become increasingly important. However, viewing unemployment as a medical issue also generates new problems [17]. Particularly vulnerable are unemployed individuals who have exhausted their maximum amount of payable sickness allowance days, but whose disability benefit applications have been rejected. It has further been suggested that conditionality may contribute to the medicalisation of unemployment and lead individuals to apply for sickness benefits in situations where employment opportunities are limited [17]. Nevertheless, the findings of this study suggest that sickness benefits may, in fact, be underutilised among the unemployed people.

### Strengths and limitations

The study used large, fully representative register data with no loss to follow-up, no missing information, and no self-report bias. Information on both the onset and duration of sickness allowance was available. However, not all work disability problems are diagnosed and therefore captured in the registers. Conversely, not all recorded diseases necessarily result in work disability and sickness absence. The data did not include information on activation obligations and only limited information on labour market programme participation. Although a wide range of covariates was included, not all relevant factors could be accounted for in the between-individual models. In the within-individual analyses, time-varying factors, such as possible changes in health status, could not be accounted for. In addition, the study population was selected in several ways and likely consists of relatively healthier individuals with stronger labour market attachment. The study population included only individuals with at least one employment or unemployment spell during the study period and excluded those with sickness allowance days in the preceding year. However, this restriction does not fully rule out the possibility that some participants reached the maximum amount of payable allowance days during the follow-up. The same applies to transitions into retirement during the follow-up period. Retirees were excluded from the study population, but some individuals may have retired during the follow-up. The findings primarily pertain to the Nordic social insurance context. Differences in insurance provisions across countries and welfare systems limit the direct comparability of results [34]. However, the mechanisms observed in this study are not unique to this context, as earlier research suggests that non-take-up of benefits and access barriers are widespread across different countries and welfare systems [21].

### Policy implications and future research

Unemployed individuals should be informed of their entitlement to sickness benefits, particularly of the long-term advantages [21]. However, this information alone is insufficient: physicians must also have adequate knowledge of these entitlements to ensure that eligible individuals are appropriately guided toward the benefits and services they need. Yet few physicians report access to consistent workplace guidelines for assessing sickness absence, and many prefer to transfer responsibility for such assessments to occupational health services [30]

Unemployed individuals should be guaranteed regular screening of work ability, improved access to healthcare and rehabilitation services, and better coordination between employment services and work-ability support systems. More suitable indicators than sickness-absence days may be needed to assess work disability among the unemployed. Decisions on rehabilitation and disability pensions may also need to consider employment status, rather than being based solely on the same criteria as for those who are employed.

Future research should examine whether participation in activation measures is associated with transitions into sickness absence by triggering such transitions, revealing health‑related work limitations, or alternatively supporting improved health [25] and reduced sickness absence.

## Conclusion

Unemployment is associated with the lower likelihood of entering sickness allowance. Its relationship with the overall prevalence of sickness allowance is more complex and appears minimal, whereas unemployment is associated with a higher total number of sickness allowance days. The findings may reflect non-take-up of sickness allowance among relatively healthier unemployed individuals with some labour market attachment.

At the same time, stricter access to disability pensions and increased obligations for unemployed people to participate in activation measures have placed those with health problems in an increasingly vulnerable position. This points to a structural contradiction within the system: applying for sickness allowance is more difficult for unemployed people than for those who are employed. In addition, employed people typically benefit from more structured processes for assessing and monitoring work ability, whereas unemployed individuals largely lack such support. Despite this, decisions concerning rehabilitation and disability pensions are often made as if information on work ability were equally comprehensive for both groups.

These findings underline the importance of reducing barriers to sickness allowance applications among unemployed people, improving the clarity and consistency of work ability assessments, and strengthening the monitoring and support of work ability for those who are unemployed.

## Data Availability

The data supportinh the findings of this study are available from Findata and Statistics Finland but restrictions apply to the availability of these data, which were used under license for the current study, and so are not publicly available. Data on the National Institute for Health and Welfare, the Finnish Center for Pensions, and the Social Insurance Institution of Finland can be accessed upon application to the centralised data permit authority Findata (https://www.findata.fi/en/, email: info@findata.fi). Data from Statistics Finland can be accessed upon application to from Statistics Finland (https://stat.fi/en/services/services-for-researchers, email: tutkijapalvelut@stat.fi).

## Abbreviations

FE: Fixed effect
IRR: Incidence rate ratio
LPM: Linear probability model
OR: Odds ratio
RD: Risk difference
RE: Random effect

## References

[1] Eurostat. Unemployment statistics. 2025. https://ec.europa.eu/eurostat/statistics-explained/index.php?title=Unemployment_statistics. Accessed 18 Feb 2026.

[2] Holmqvist M. The ‘active welfare state’ and its consequences: a case study of sheltered employment in Sweden. Eur Soc. 2010;12:209–30. 10.1080/14616690903388960.

[3] Horn A, Kevins A, van Kersbergen K. The paternalist politics of punitive and enabling workfare: evidence from a new dataset on workfare reforms in 16 countries, 1980–2015. Socioecon Rev. 2023;21:2137–66. 10.1093/ser/mwac060.

[4] Ylistö S, Husu H-M. Negative emotional consequences of labour market activation policies for long-term unemployed young adults in Finland. Int J Sociol Soc Policy. 2021;41:1–15. 10.1108/IJSSP-02-2021-0039.

[5] Böckerman P, Ilmakunnas P. Unemployment and self-assessed health: evidence from panel data. Health Econ. 2009;18:161–79. 10.1002/hec.1361. 18536002 10.1002/hec.1361

[6] McKee-Ryan F, Song Z, Wanberg CR, Kinicki AJ. Psychological and physical well-being during unemployment: a meta-analytic study. J Appl Psychol. 2005;90:53–76. 10.1037/0021-9010.90.1.53. 15641890 10.1037/0021-9010.90.1.53

[7] Paul KI, Moser K. Unemployment impairs mental health: meta-analyses. J Vocat Behav. 2009;74:264–82. 10.1016/j.jvb.2009.01.001.

[8] Schmitz H. Why are the unemployed in worse health? The causal effect of unemployment on health. Labour Econ. 2011;18:71–8.

[9] Silver SR, Li J, Quay B. Employment status, unemployment duration, and health-related metrics among US adults of prime working age: Behavioral Risk Factor Surveillance System, 2018-2019. Am J Ind Med. 2022;65:59–71. 10.1002/ajim.23308. 34748231 10.1002/ajim.23308 PMC8678322

[10] Junna L, Moustgaard H, Martikainen P. Health-related selection into employment among the unemployed. BMC Public Health. 2022;22:657. 10.1186/s12889-022-13023-0. 35382786 10.1186/s12889-022-13023-0 PMC8985275

[11] Roelfs DJ, Shor E, Davidson KW, Schwartz JE. Losing life and livelihood: a systematic review and meta-analysis of unemployment and all-cause mortality. Soc Sci Med. 2011;72:840–54. 10.1016/j.socscimed.2011.01.005. 21330027 10.1016/j.socscimed.2011.01.005 PMC3070776

[12] Pharr JR, Moonie S, Bungum TJ. The impact of unemployment on mental and physical health, access to health care and health risk behaviors. Int Sch Res Not. 2011;2012:e483432. 10.5402/2012/483432.

[13] Blomgren J, Jäppinen S. Incidence and length of sickness absence among hierarchical occupational classes and non-wage-earners: A register study of 1.6 million Finns. Int J Environ Res Public Health. 2021;18:501. 10.3390/ijerph18020501. 33435424 10.3390/ijerph18020501 PMC7827837

[14] Blomgren J, Perhoniemi R. Long-term sickness absence in Finland : trends by sex, age and diagnostic group from 2010–2023. Helsinki: Kela; 2025.

[15] Harkko J, Virtanen M, Kouvonen A. Unemployment and work disability due to common mental disorders among young adults: selection or causation? Eur J Public Health. 2018;28:791–7. 10.1093/eurpub/cky024. 29514230 10.1093/eurpub/cky024

[16] Helgesson M, Johansson B, Nordqvist T, Lundberg I, Vingård E. Unemployment at a young age and later sickness absence, disability pension and death in native Swedes and immigrants. Eur J Public Health. 2013;23:606–10. 10.1093/eurpub/cks099. 22930745 10.1093/eurpub/cks099 PMC3719474

[17] Linden P, Reibling N. Medicalisation of unemployment: an analysis of sick leave for the unemployed in Germany using a three-level model. Work Employ Soc. 2025;39:139–62. 10.1177/09500170241244688.

[18] Sickness allowance. Kela. 2026. https://www.kela.fi/sickness-allowance. Accessed 18 Feb 2026.

[19] Goedemé T, Janssens J. The concept and measurement of non-take-up. An overview, with a focus on the non-take-up of social benefits. Deliverable 9.2, InGRID. Leuven: Deliverable 9.2, InGRID; 2020.

[20] van Oorschot WJH. Modelling non-take-up: The interactive model of multi- level influences and the dynamic model of benefit receipt. In: van Oorschot WJH, editor. New perspectives on the non-take-up of social security benefits. Tilburg: Tilburg University Press; 1996.

[21] Janssens J, Van Mechelen N. To take or not to take? An overview of the factors contributing to the non-take-up of public provisions. Eur J Soc Secur. 2022;24:95–116. 10.1177/13882627221106800.

[22] Eronen A, Landén P, Palanne A, Peltosalmi J, Teittinen A. Sosiaalibarometri 2025. Helsinki: Soste; 2025.

[23] Rinne H, Sihvonen A-P, Väisänen V, Leemann L, Aalto A-M. Unmet need for healthcare services among unemployed people – findings from a national survey in Finland. BMC Health Serv Res. 2025;25:1616. 10.1186/s12913-025-13765-8. 41462257 10.1186/s12913-025-13765-8 PMC12751575

[24] Linden P, Reibling N. Unemployed + sick = more deserving? A survey experiment on how the medicalization of unemployment affects public opinion. Front Sociol. 2022. 10.3389/fsoc.2022.738397. 35602003 10.3389/fsoc.2022.738397 PMC9120940

[25] Puig-Barrachina V, Giró P, Artazcoz L, Bartoll X, Cortés-Franch I, Fernández A, et al. The impact of Active Labour Market Policies on health outcomes: a Scoping review. Eur J Public Health. 2020;30:36–42. 10.1093/eurpub/ckz026. 30907412 10.1093/eurpub/ckz026

[26] Klavus J, Forma L, Partanen J, Rissanen P. Need and utilization of primary health care among long-term unemployed Finns. J Health Soc Sci. 2018;3:231–42.

[27] Virtanen P, Kivimäki M, Vahtera J, Koskenvuo M. Employment status and differences in the one-year coverage of physician visits: different needs or unequal access to services? BMC Health Serv Res. 2006;6:123. 10.1186/1472-6963-6-123. 17014702 10.1186/1472-6963-6-123 PMC1618837

[28] Rinne H, Blomgren J. Use of outpatient healthcare services before and after the onset of unemployment: a register-based propensity score matched study from Finland. PLoS ONE. 2023;18:e0288423. 10.1371/journal.pone.0288423. 37556479 10.1371/journal.pone.0288423 PMC10411812

[29] Hinkka K, Niemelä M, Autti-Rämö I, Palomäki H. Physicians’ experiences with sickness absence certification in Finland. Scand J Public Health. 2019;47:859–66. 10.1177/1403494818758817. 29485317 10.1177/1403494818758817

[30] Heikkilä E, Saastamoinen P, Cansel A, Mikkonen J, Vänskä J, Leskelä R-L. Sairauspoissaolojen arvioinnissa on yhä vaikeuksia [One-third of doctors that answered the survey find sick leave describing still problematic]. Lääkärilehti. 2024;79:e38866. https://www.laakarilehti.fi/e38866.

[31] Winde LD, Alexanderson K, Carlsen B, Kjeldgård L, Wilteus AL, Gjesdal S. General practitioners’ experiences with sickness certification: a comparison of survey data from Sweden and Norway. BMC Fam Pract. 2012;13:10. 10.1186/1471-2296-13-10. 22375615 10.1186/1471-2296-13-10 PMC3320536

[32] Ljungquist T, Hinas E, Nilsson GH, Gustavsson C, Arrelöv B, Alexanderson K. Problems with sickness certification tasks: experiences from physicians in different clinical settings. A cross-sectional nationwide study in Sweden. BMC Health Serv Res. 2015;15:321. 10.1186/s12913-015-0937-6. 26264627 10.1186/s12913-015-0937-6 PMC4533961

[33] Ministy of Social Affairs. Kuntoutuksen uudistamiskomitean ehdotukset kuntoutusjärjestelmän uudistamiseksi. Helsinki: Sosiaali- ja terveysministeriö; 2017.

[34] Hensing G. The measurements of sickness absence – a theoretical perspective. Norsk Epidemiologi. 2009;19. 10.5324/nje.v19i2.584.

[35] Lahtinen H, Sirniö O, Martikainen P. Social class and the risk of unemployment: trends, gender differences and the contribution of education. Acta Sociol. 2020;63:303–21. 10.1177/0001699318817594.

[36] Saastamoinen LK, Aaltonen K, Maljanen T, Tuominen U, Martikainen JE, editors. Health registers as a source of data for research and policy making. Dosis. 2012;28:199–205.

[37] Ruottunen S. Can activation policies foster sustainable wellbeing? Phenomenological analysis of long-term unemployed jobseekers’ lived experiences. J Soc Policy. 2026; 1–19. 10.1017/S0047279426101330.

[38] Hultin H, Lindholm C, Möller J. Is there an association between long-term sick leave and disability pension and unemployment beyond the effect of health status? – a cohort study. PLoS ONE. 2012;7:e35614. 10.1371/journal.pone.0035614. 22558176 10.1371/journal.pone.0035614 PMC3338415

[39] Perhoniemi R, Blomgren J, Laaksonen M. Unemployed and disabled for work: identifying 3-year labour market pathways from the beginning of a sickness absence using sequence and cluster analyses in a register-based longitudinal study in Finland. BMJ Open. 2023;13:e076435. 10.1136/bmjopen-2023-076435. 38151282 10.1136/bmjopen-2023-076435 PMC10753752

[40] Støver M, Pape K, Johnsen R, Fleten N, Sund ER, Claussen B, et al. Unemployment and disability pension-an 18-year follow-up study of a 40-year-old population in a Norwegian county. BMC Public Health. 2012;12:148. 10.1186/1471-2458-12-148. 22369630 10.1186/1471-2458-12-148 PMC3305666

[41] Laaksonen M, Blomgren J. The level and development of unemployment before disability retirement: a retrospective study of Finnish disability retirees and their controls. Int J Environ Res Public Health. 2020;17:1756. 10.3390/ijerph17051756. 32182683 10.3390/ijerph17051756 PMC7084885

[42] Gould R, Härkäpää K, Järvikoski A. Toimiiko työeläkekuntoutus? 2012. https://www.julkari.fi/handle/10024/129345. Accessed 17 Jan 2024.

[43] Laaksonen M, Nyman H. Työkyvyttömyyseläkkeiden hylkäysosuuden kasvu 2007–2016. 2018. https://www.julkari.fi/handle/10024/137211. Accessed 17 Jan 2024.

